# Steady-State Motion Visual Evoked Potential (SSMVEP) Based on Equal Luminance Colored Enhancement

**DOI:** 10.1371/journal.pone.0169642

**Published:** 2017-01-06

**Authors:** Wenqiang Yan, Guanghua Xu, Min Li, Jun Xie, Chengcheng Han, Sicong Zhang, Ailing Luo, Chaoyang Chen

**Affiliations:** 1 School of Mechanical Engineering, Xi’an Jiaotong University, Xi’an, China; 2 State Key Laboratory for Manufacturing Systems Engineering, Xi’an Jiaotong University, Xi’an, China; 3 Department of Biomedical Engineering, Wayne State University, Detroit, Michigan, United States of America; National University of Defense Technology College of Mechatronic Engineering and Automation, CHINA

## Abstract

Steady-state visual evoked potential (SSVEP) is one of the typical stimulation paradigms of brain-computer interface (BCI). It has become a research approach to improve the performance of human-computer interaction, because of its advantages including multiple objectives, less recording electrodes for electroencephalogram (EEG) signals, and strong anti-interference capacity. Traditional SSVEP using light flicker stimulation may cause visual fatigue with a consequent reduction of recognition accuracy. To avoid the negative impacts on the brain response caused by prolonged strong visual stimulation for SSVEP, steady-state motion visual evoked potential (SSMVEP) stimulation method was used in this study by an equal-luminance colored ring-shaped checkerboard paradigm. The movement patterns of the checkerboard included contraction and expansion, which produced less discomfort to subjects. Feature recognition algorithms based on power spectrum density (PSD) peak was used to identify the peak frequency on PSD in response to visual stimuli. Results demonstrated that the equal-luminance red-green stimulating paradigm within the low frequency spectrum (lower than 15 Hz) produced higher power of SSMVEP and recognition accuracy than black-white stimulating paradigm. PSD-based SSMVEP recognition accuracy was 88.15±6.56%. There was no statistical difference between canonical correlation analysis (CCA) (86.57±5.37%) and PSD on recognition accuracy. This study demonstrated that equal-luminance colored ring-shaped checkerboard visual stimulation evoked SSMVEP with better SNR on low frequency spectrum of power density and improved the interactive performance of BCI.

## Introduction

Commonly used brain-computer interface (BCI) technologies include motor imagery (MI) [1], P300 event-related potential (ERP) [2], transient visual evoked potential (tVEP) [3], and steady-state visual evoked potential (SSVEP) [4]. Compared with other methods, the SSVEP requires less electrodes for electroencephalography (EEG) recording and no need for training, and can obtain higher recognition accuracy. However, it usually provides stimulation through light flicking or graphic flipping, which is likely to cause visual fatigue and discomfort with a consequent decrease of recognition accuracy. In recent years, brain-computer interface paradigms based on motion perception have been proposed [5] to avoid the negative influence of prolonged strong stimulation on brain neurons. Motion visual evoked potentials (mVEPs) can be divided into transient ones and steady-state ones [6]. In 2009, Gao et al. adopted transient N2 potential based on the perception of motion [7],[8]. This paradigm has an obvious advantage in VEP-based BCI study in which constant luminance and non-flashing techniques were used. However, its shortcoming is that the transient paradigm requires multiple stimulation targets to move along a single direction resulting in motion after-effect (MAF) [9]. Xie et al. designed a BCI paradigm with Newton's rings based on steady-state motion visual evoked potential [10] that increased recognition accuracy to a favorable level [11]. However, the luminance in the central area of the Newton's ring can not be kept constant during the process of motion resulting in a lower signal-to-noise ratio (SNR) of spectral peaks.

**Fig 1:**
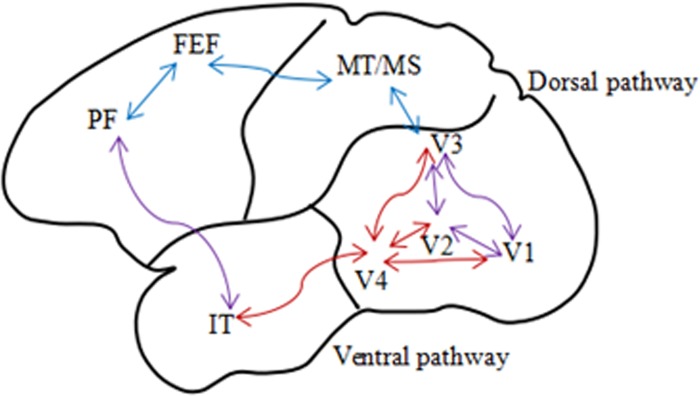
Pathways of human visual cortex.

SSVEP mainly uses light intensity sensing pathways of human vision. Human visual system (HVS) is elaborate and sophisticated. It is composed of retina [12], lateral geniculate nucleus (LGN), and visual cortex. Visual cortex is the high-level central neural system, which includes striate cortex V1 and extra striate cortex (e.g. V2, V3, V4 and V5 (MT)). As shown in Fig 1, primary visual cortex V1 has two pathways to output information: dorsal pathway and ventral pathway [13].

The dorsal pathway, also known as M-pathway, is associated with motion recognition and spatial processing, which detects motion speed and direction. The ventral pathway, also known as P-pathway, is a color-sense-associated and object recognition pathway, which detects luminance and color [14], [15]. When two different colors with the same luminance are applied for visual stimulation, the sensitivity of the eyes against flicker will be reduced to the lowest [16],[17],[18].

Our hypothesis is that equal-luminance colored checkerboard visual stimulation activates more neuronal response in visual center and M- and P-pathways in the brain with a higher SNR of SSMVEP signals. A colored ring-shaped checkerboard composed of color, shape, luminance, and motion was designed in this study according to neural mechanisms of HVS. The purpose of this study was to determine if the equal-luminance colored ring-shaped checkerboard stimulation paradigm evokes distinct SSMVEPs with a better SNR, and improves the interactive performance of BCI.

## Materials and Methods

### Ethics statement

Subjects were studied after giving informed written consent in accordance with a protocol approved by the institutional review board of Xi'an Jiaotong University.

### Design of equal-luminance colored checkerboard paradigm

**Fig 2:**
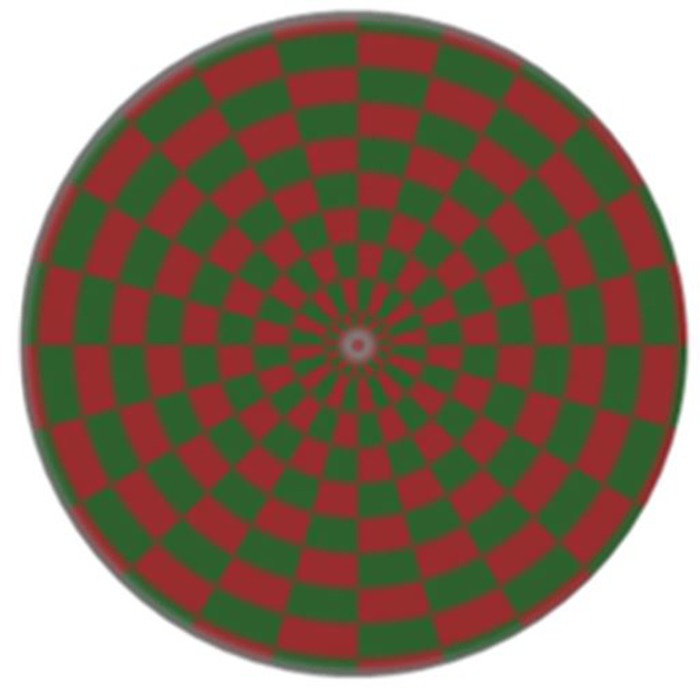
Pattern of checkerboard paradigm. The coordinate (*x*, *y*) in the formulas (1) and (2) refer to the coordinate position of each pixel of the pattern on the display screen.

The ring-shaped checkerboard was divided into small grids with the same size and number, with two different colors arranged alternatively. The luminance at the central part of stimulation units was set to be the background luminance value to ensure constant luminance during contraction-expansion of the checkerboard. A white spot with radius of 1 pixel was set at the center to keep the subjects focus on it during the experiment. Based on the color space theory, three pairs of antagonism colors—black and white, red and green, and blue and yellow—are suitable for color stimulation to ensure accurate transmission of colors [19]. Black and white mainly represent eye-to-luminance reaction. Blue and yellow have relatively poor robustness, red and green can present images under equal luminance. Thus, red and green were selected as the colored stimulation paradigm in this study, and the paradigm pattern is as shown in Fig 2.

The formula for generating the stimulation pattern of ring-shaped checkerboard is:

$$I=\{\begin{array}{c} sign(x)+I_{0},r>R_{inner}orr<R_{outer}\\ I_{0},other \end{array},$$ (1)

where *sign*(*x*) is a sign function:

$$sign\left(x\right)=sign\left\{\cos\left[\pi \frac{r\left(x,y\right)}{D}+\phi \left(t\right)\frac{L}{D}\right]\cdot \cos\left[ang\left(x,y\right)\cdot M\right]\right\},$$ (2)

*r*(*x*,*y*) and *ang*(*x*,*y*) are the radius and angle of the pattern pixel point (*x*,*y*) on the display screen; *D* is the width of the checkerboard, representing spatial resolution, and it was set to be 10 pixels, with the checkerboard divided into 8 rings; *ϕ*(*t*) is the phase value function; *L* is the motion amplitude of the checkerboard, and it was set to be 10 pixels; *M* is the number of checks which are cut from a single ring, and it was set to be 12 pixels; *I*_*0*_ is the background luminance, and it was set to be 120 pixels; *R*_*inner*_ and *R*_*outer*_ are inner and outer diameters of the checkerboard, and they were set to be 3 pixels and 80 pixels.

Sine was adopted to show the contraction-expansion of the checkerboard, where:

$$\phi \left(t\right)=\frac{\pi }{2}+\frac{\pi }{2}\cdot \sin\left(2\pi \cdot f_{c}\cdot t-\frac{\pi }{2}\right),$$ (3)

*f*_*c*_ is the motion frequency, i.e. the reciprocal of the required time for one time of contraction-expansion of the checkerboard. When the phase *ϕ*(*t*) is turned from 0 to π, the checkerboard contracts, and when the phase *ϕ*(*t*) is turned from π to 0, the checkerboard expands (The specific forms of movement can refer to the S1 Video). Motion direction changes twice in one cycle. The frequency of motion direction changes is defined to be motion inversion frequency *f*, and it is 2 times of motion frequency *f*_*c*_. Since SSMVEP mainly comes from brain activities which triggered by direction changes, and the energy mainly focused on motion inversion frequency. Therefore, the inversion frequency is adopted as the fundamental frequency of visual stimulation in this study.

In this study, visual stimulation was presented to subjects through the computer screen, and screen refresh rate was *f*_*r*_. To generate frame images, the *t* in Formula (3) must be discredited according to the screen refresh rate, i.e. *t*(*n*) *= n / f*_*r*_, where *n =* 1,2,3…… is the frame number. Formula (3) can be adapted to:

$$\phi \left(n\right)=\frac{\pi }{2}+\frac{\pi }{2}\cdot \sin\left(2\pi \cdot n\cdot \frac{f_{c}}{f_{r}}-\frac{\pi }{2}\right).$$ (4)

Phase value function *ϕ*(*n*) is a discrete time sequence. In order to ensure *ϕ*(*n*) is a periodic sequence, the value of *f*_*r*_ */ f*_*c*_ must be an integer. To command *F*_*C =*_ *f*_*r*_ */ f*_*c*_ to be frames required for one cycle of expansion-contraction motion. Formula (4) is rewritten as:

$$\phi \left(n\right)=\frac{\pi }{2}\cdot \sin\left(\frac{2\pi n}{f_{c}}-\frac{\pi }{2}\right).$$ (5)

Thus, the calculation formula of motion inversion frequency is:

$$f=\frac{2f_{r}}{F_{c}}.$$ (6)

When appropriate *F*_*C*_ is chosen in actual application, a correct motion inversion frequency *f* can be calculated based on Formula (6), and the phase value function *ϕ*(*n*) can be calculated based on Formula (5).

### Experiment data processing

Signals collected from each subject were analyzed off-line. If stimulation lasts *t* seconds per trial, *n* trials in total, the sampling frequency of electroencephalogram (EEG) equipment was *F*_*s*_. EEG segment for each stimulation was extracted based on the starting and ending points in each trial, so a n×t*F_*s*_ dimensional matrix was obtained. Then stimulation signals in all trials were superposed and averaged to obtain a new data segment *X* = {*x*(*1*), *x*(*2*),…*x*(*i*)}, *i* = 1,2,3,…,t*F_*s*_. Then the new data segment was processed by 4–40 Hz band-pass filtering to remove low-frequency drift and high-frequency interference. Then the data segment was analyzed using Welch power spectrum density [20] mentioned below.

### Welch Power spectrum density

In the BCI field, SNR is a key index to evaluate the efficiency of stimulation paradigms. A good stimulation paradigm should evoke more powerful SSMVEP, which indicated in the power spectrum is that the peak value is higher at corresponding stimulation frequency.

In this study, the Welch power spectral density was used to estimate random signals by dividing a data with a length of *N* into *M* segments, and the length of each segment is *l*. Its window averaged period formula is:

$$p\left(w\right)=\frac{1}{M}\sum_{i=1}^{M}\left(\frac{1}{lp_{0}}{\left|\sum_{n=1}^{l}w\left(n\right)x_{i}\left(n\right)e^{-jwn}\right|}^{2}\right),$$ (7)

where *p*_*0*_ refers to the power of window *w(n)*:

$$p_{0}=\frac{1}{l}{\sum_{n=1}^{l}\left|w\left(n\right)\right|}^{2}.$$ (8)

### Canonical correlation analysis

Canonical correlation analysis (CCA) is widely applied in SSMVEP target recognition. In this study, EEG of all sampling channels were selected as a set of variable to calculate canonical correlation coefficient with the generated reference signals. The maximum target of correlation coefficient is considered to be the focused target [10]. The SSMVEPs collected from positions of designated electrodes were marked as *X* = (*x*_*1*,_ *x*_*2*,…,_*x*_*n*_), where *n* is the number of electrode channels. The reference signals were constructed at the stimulation frequency *f*_*i*_:

$$Y_{i}=\left\{\begin{array}{l} \cos\left(2\pi \cdot f_{i}\cdot t\right)\\ \sin\left(2\pi \cdot f_{i}\cdot t\right)\\ \;\vdots \\ \cos\left(2\pi \cdot kf_{i}\cdot t\right)\\ \sin\left(2\pi \cdot kf_{i}\cdot t\right) \end{array}\right\},t=\frac{1}{f_{s}},\cdots ,\frac{m}{f_{{}_{s}}},$$ (9)

Where *k* is the number of harmonics, which is dependent on how many frequency harmonics existed in SSMVEP. The *f*_*s*_ is sampling rate, and *m* is sample points. By calculating:

$$\rho_{i}=\frac{E\left(W_{x}{}^{T}XY_{i}{}^{T}W_{y_{i}}\right)}{\sqrt{E\left(W_{x}{}^{T}XX^{T}W_{x}\right)\cdot E\left(W_{y_{i}}{}^{T}Y_{i}Y_{i}{}^{T}W_{y_{i}}\right)}}.$$ (10)

Correlation coefficient *ρ*_*i*_ can be obtained, and *i* corresponding to the maximum is the focused target.

### Feature recognition algorithm based on power spectrum density peak

If the equal-luminance red-green checkerboard can increase higher SNR, a significant peak will appear on the power spectrum density at the stimulation frequency. In consideration of these circumstances, a characteristics recognition method based on power spectrum density peak was used in this study.

Several stimulation paradigms with different stimulation frequency were displayed on the screen, and we assumed that the stimulation frequency of all paradigms have a minimum of *f*_*min*_ and a maximum of *f*_*max*_. The stimulation duration per trial was set as *t* seconds and the EEG sampling frequency as *F*_*s*_. Subjects stared on one of the stimulation paradigms, and the data sequence of every sampling channel were {*X*_*f*_ (*1*), *X*_*f*_ (*2*),…, *X*_*f*_ (*n*)}. First, singular value decomposition (SVD) was performed for each channel data to eliminate noise. Then Welch power spectrum density was calculated for every channel data to find out the maximum peak {*p*_*1*_(*w*), *p*_*2*_(*w*),…, *P*_*n*_(*w*)} within the [*f*_*min*_, *f*_*max*_]. Based on *p*_*1*_*(w)*, the weight coefficient of each channel was calculated, i.e. *a*_*1*_ = 1, *a*_*2*_ = *p*_*2*_(*w*)/*p*_*1*_(*w*),…, *a*_*n*_ = *p*_*n*_(*w*)/*p*_*1*_(*w*). Thus the weight coefficient of each channel was obtained {*a*_*1*_, *a*_*2*_,…, *a*_*n*_}.

The data of the *n* channels was multiplied by weight coefficient, then superposed and averaged to get a new data vector:

$$x=\frac{1}{n}\sum_{i=1}^{n}\left(X\left(i\right)\cdot a_{i}\right).$$ (11)

The data size is 1×t*F_*s*_. This method that makes the multi-electrode signals merge into a single channel signal called spatial filtering. In the EEG signal processing, integration of multi-electrode EEG information is effective [21],[22]. After such processing, noises were suppressed, which made stimulation frequency peak outstanding. The Welch power spectrum density was then calculated. The *idx* of the data point was identified corresponding to the maximum peak within the [*f*_*min*_-Δ*f*, *f*_*max*_+Δ*f*], and located on the corresponding x-coordinate, which was identical to the stimulation frequency *f*. Where Δ*f* is frequency difference, that was set as 0.2 Hz.

The difference between the obtained stimulation frequency *f* and that of all paradigms was calculated to figure out the absolute value of the difference:

$$\Delta f_{i}=\left|f-f_{i}\right|,i=1,2,\cdots ,n,$$ (12)

*i* refers to the *i* stimulation paradigm. When Δ*f*_*i*_ is the minimum, the corresponding *i* is the focused target.

### BCI experiment platform

**Fig 3:**
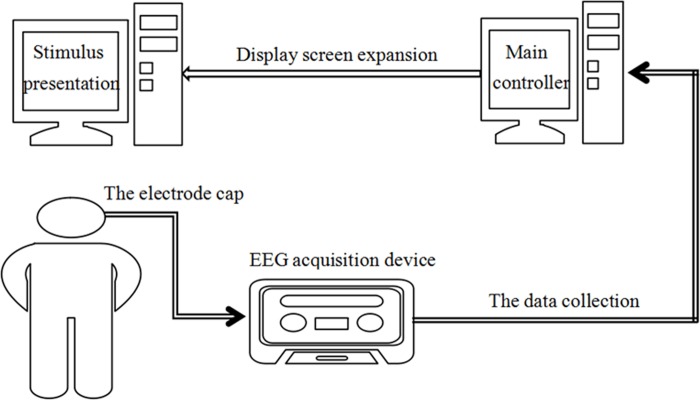
BCI lab table.

**Fig 4:**
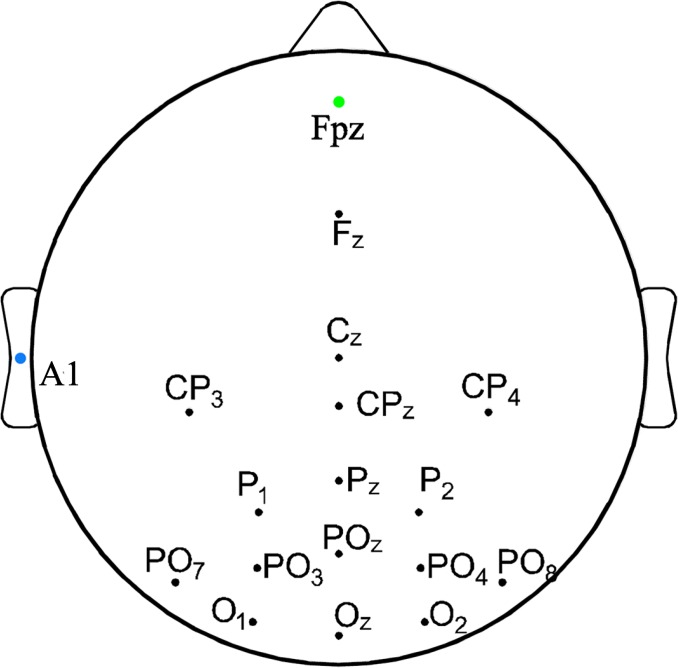
Configuration of electrode locations used in this study.

In this study, a g.USBamp (g.tec, Austrian) was used to collect and process EEG. This equipment can collect signals of 16 channels at the same time, with sampling frequency of 1,200 Hz. EEG amplifier g.USBamp and active electrode system g.GAMMAbox were combined to form a BCI experiment platform, as shown in Fig 3. Before the experiment, the reference electrode A1 was placed at the left ear of the subjects, and the ground electrode Fpz was placed at forehead. EEG was also collected from the following 15 channels: O1, Oz, O2, PO7, PO3, POz, PO4, Cz, P1, Pz, P2, CP3, CPz, CP4 and Fz, with electrodes places as shown in Fig 4.

### Experiment of brain response enhancement under equal-luminance colored stimulation

**Fig 5:**
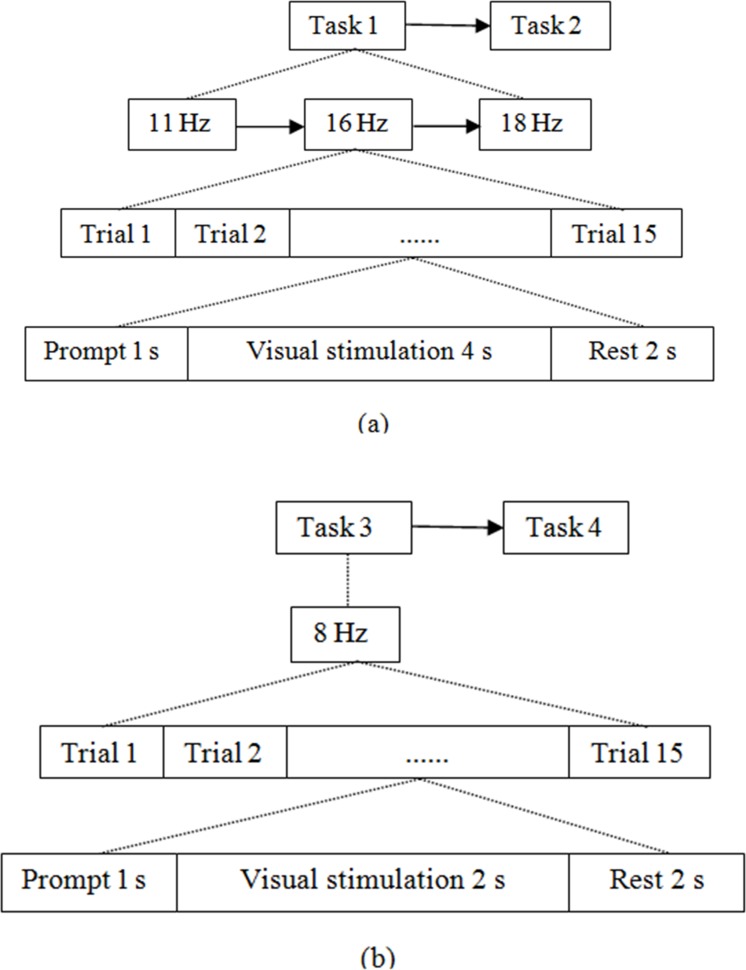
The process of experiment (a). (a) The experiment process of task 1 and task 2. (b) The experiment process of task 3 and task 4.

A black-white checkerboard was used for luminance calibration, which doesn't contain color information. Total luminance value of black-white and equal-luminance red-green checkerboard were the same, which was set as 76 *cd/m*^*2*^. We used 5 males and 4 females (20–25 years old) as subjects, who have normal color and visual senses. The experimental process is shown in Fig 5(A) and 5(B). Each subject was requested to finish four tasks. Task 1, black-white checkerboard stimulation: Three stimulation paradigms were displayed on the screen. The frequencies were 11 Hz, 16 Hz and 18 Hz respectively. Subjects were requested to stare on one of the paradigms for 4 s per trial, 15 trials in total, with an interval of 2 s, and then stared on another paradigm, until all were done. Task 2, equal-luminance red-green checkerboard stimulation: Experimental contents were same as Task 1. Task 3, black-white checkerboard stimulation: One stimulation paradigm was displayed on the screen, and the frequency was 8 Hz. Stimulation lasts 2 s per trial, 15 trials in total, with an interval of 2 s. Task 4, equal-luminance red-green checkerboard stimulation: Experimental contents were same as Task 3. The experiments were designed to compare differences of brain response evoked by equal-luminance red-green and black-white checkerboard to determine the feasibility of improving BCI interactive performance using equal-luminance colored stimulation.

### Comparisons of experimental stimulation for recognition accuracy between black-white and equal-luminance red-green checkerboard

**Fig 6:**
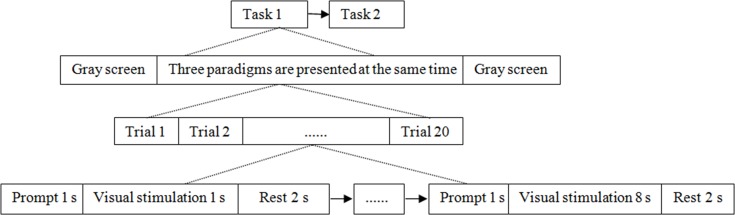
The process of experiment (b).

Nine people in experiment (a) were selected as subjects. The experimental process is shown in Fig 6. Task 1, black-white checkerboard stimulation: Three stimulation paradigms were displayed on the screen, and the stimulation frequencies were 8 Hz, 9 Hz and 10 Hz respectively. Subjects stared on any of them every trial. The stimulation duration per trial was divided into eight levels, from 1 s to 8 s, and every subject stared on 20 trials in each level of experiments, with an interval of 2 s. Task 2, equal-luminance red-green checkerboard stimulation: Experimental contents were same as Task 1. CCA was used to identify fixation target of each trial.

### Characteristic recognition experiment based on power spectrum density peak

**Fig 7:**
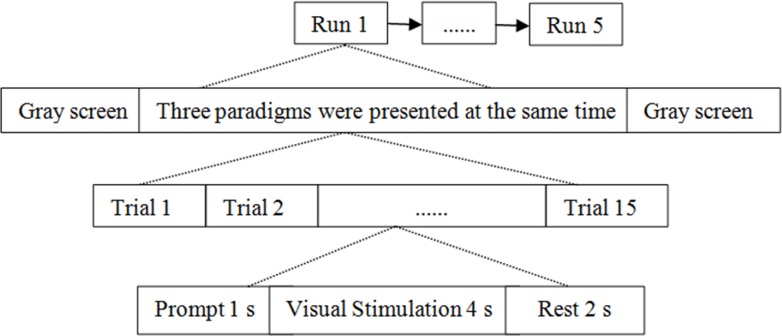
The process of experiment (c).

Equal-luminance red-green checkerboard was used as stimulus paradigm. Nine people in experiment (a) were selected as subjects. Three stimulation paradigms were displayed on the screen, and the stimulation frequencies were 10.5 Hz, 11.5 Hz, and 12.5 Hz respectively. The experimental process is shown in Fig 7. The subjects stared on any of the paradigms for 4 s per trial, 15 trials in total, with an interval of 2 s. Recognition accuracy result of each trial by both CCA and PSD was analyzed. In order to get the statistical analysis of the accuracy of each subject, the above experiments were carried out 5 runs.

## Results

### Experimental Results of brain response enhancement under equal-luminance colored stimulation

#### Brain response enhancement under colored stimulation

**Fig 8:**
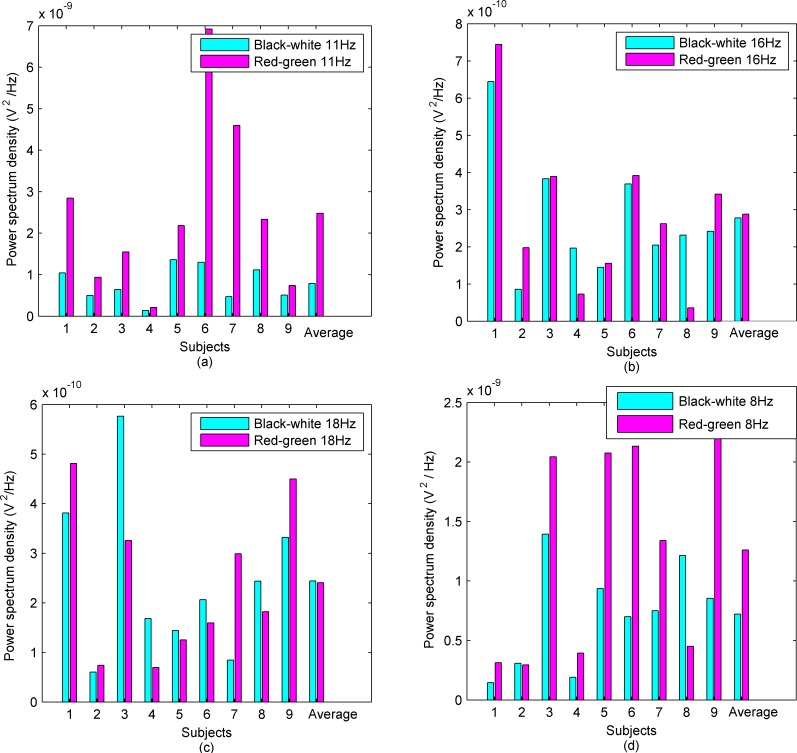
Average power spectrum density of subjects at different stimulation frequencies. (a) Average power spectrum density of subjects at 11 Hz. Equal-luminance red-green checkerboard induced higher EEG SNR. (b) Average power spectrum density of subjects at 16 Hz. The differences of the two paradigms are not significant. (c) Average power spectrum density of subjects at 18 Hz. The differences of the two paradigms are not significant. (d) Average power spectrum density of subjects at 8 Hz. We shortened the stimulus duration per trial to 2 seconds and equal-luminance red-green checkerboard still induced higher SNR.

The peak value of the power spectrum density of each sampling channel at a designated stimulation frequency was calculated, superposed, averaged for mean values and standard deviations (SD). Fig 8(A)–8(D) are the average power spectrum density of 9 subjects at different stimulation frequencies. Fig 8(A) shows that at 11 Hz, the equal-luminance colored checkerboard can evoke more powerful SSMVEP and higher SNR. All subjects had favorable stimulation effects by the equal-luminance red-green checkerboard. Fig 8(B) and 8(C) show that at the middle frequency spectrum (15 Hz-25 Hz), i.e. 16 Hz and 18 Hz, the equal-luminance red-green checkerboard had no significant enhancement effect when compared with the black-white checkerboard. As shown in Fig 8(D), the stimulation frequency was 8 Hz. When the stimulation duration decreased to 2 s per trial, the red-green checkerboard still had enhancement effects. Fig 8(A)–8(D) also show that brain response at the low frequency spectrum (lower than 15 Hz) was the most intensive, which was consistent with the results of literature [6].

**Fig 9:**
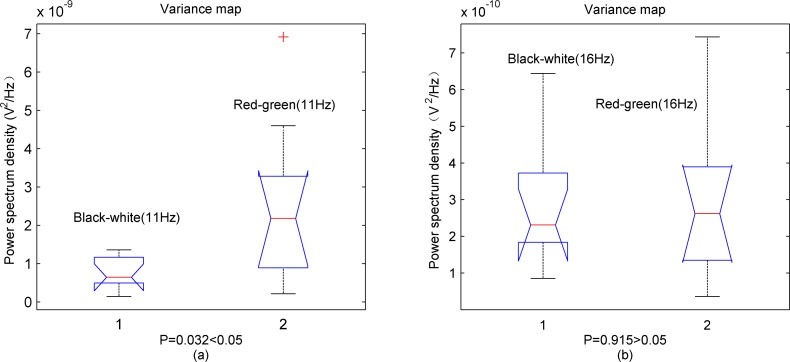
Average power spectrum density variance maps of subjects at 11 Hz and 16 Hz. (a) Average power spectrum density variance map of subjects at 11 Hz. The equal-luminance colored stimulus has a significant effect on the brain response than black-white. (b) Average power spectrum density variance map of subjects at 16 Hz. There is no significant difference on the brain response between black-white and red-green stimulus at middle frequency spectrum (16 Hz).

Designated frequency of 11 Hz in low frequency spectrum and 16 Hz in middle frequency spectrum were selected to conduct the significance test. One-way analysis of variance (ANOVA) was used for statistical analysis. With *P*<0.05 considered as a significant level. Fig 9(A) shows the variance maps for the stimulus frequency of 11 Hz and 16 Hz. The *P* value is less than 0.05 when the stimulation frequency is 11Hz. Fig 9(B) shows the *P* value is greater than 0.05 when the stimulation frequency is 16 Hz.

### Experimental Results of recognition accuracy between black-white and equal-luminance red-green checkerboard

#### Recognition accuracy of the black-white and equal-luminance red-green checkerboard

**Fig 10:**
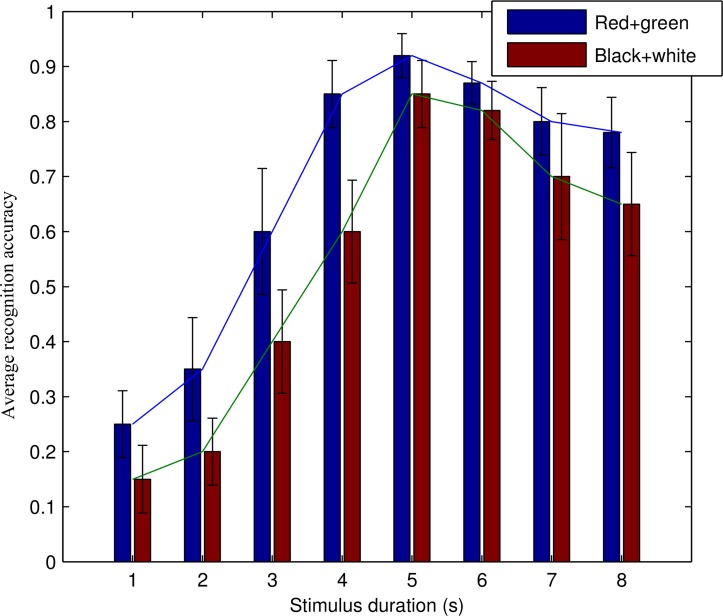
Mean variance histogram of the recognition accuracy of subjects with different stimulation duration. First, we calculated the accuracy of each subject with different stimulation duration. Then we averaged recognition accuracy of 9 subjects according to stimulation duration. The equal-luminance red-green checkerboard always has higher recognition accuracy than that of the black-white checkerboard.

CCA was used to calculate the recognition accuracy of black-white and equal-luminance red-green checkerboard for each subject. The accuracy of each subject with different stimulation duration was calculated to average the recognition accuracy of 9 subjects according to stimulation duration. Fig 10 is the mean variance histogram of the recognition accuracy, the equal-luminance red-green checkerboard always had higher recognition accuracy than that of the black-white checkerboard with different stimulation duration. When the stimulus duration was within 4 s, red-green checkerboard recognition accuracy was higher than that of the black-white checkerboard. When the stimulus duration exceeded 4 s, the difference of recognition accuracy was not greater. When the stimulation duration exceeded 6 s, subjects reported visual fatigue and the recognition accuracy decreased.

We used Chi-Square test to conduct significance test and the significant level was set as 0.05. As shown in Table 1, the recognition accuracy of equal-luminance red-green checkerboard significantly improved except when the stimulus duration were 5 s and 6 s. But the accuracy of equal-luminance red-green checkerboard was higher than black-white checkerboard when the stimulus duration were 5 s and 6 s. When the stimulus duration was 7 s or 8 s, the recognition accuracy of equal-luminance red-green checkerboard was still higher than black-white paradigm.

**Table 1:** Comparison of average recognition accuracy between Red-Green and Black-White paradigms.

Stimulus duration (s)		R-G (%)	B-W (%)	P
1	25.00		15.00	0.018
2	35.00		20.00	0.001
3	60.00		40.00	0.000
4	85.00		60.00	0.000
5	86.67		81.67	0.194
6	87.78		82.22	0.140
7	81.67		70.00	0.010
8	80.00		65.00	0.001

### Experimental Results of characteristic recognition based on power spectrum density peak

#### Recognition of power spectrum density characteristics

**Fig 11:**
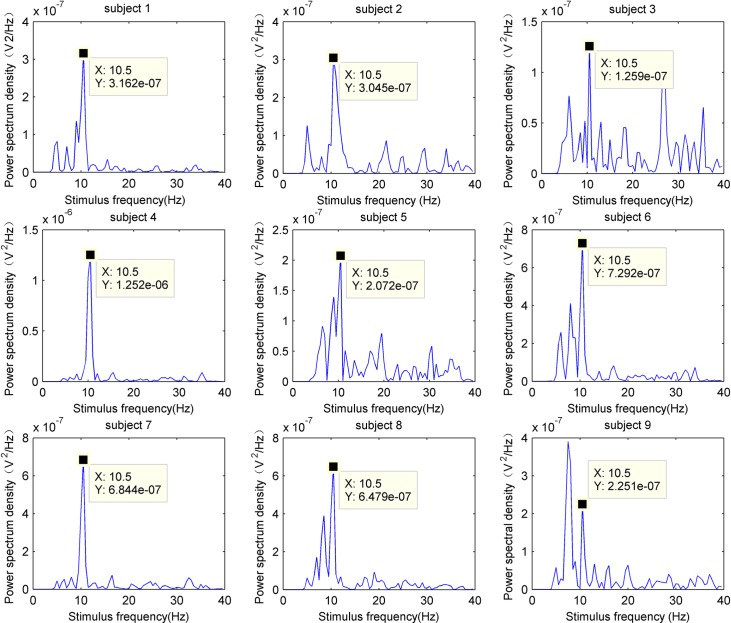
Power spectrum density of subjects at 10.5 Hz.

Fig 11 shows the power spectrum density of subjects processed by the proposed method for the first stimulation paradigm in 10.5 Hz. Most subjects had significant peaks at 10.5 Hz except subject 9, indicating that SSMVEP firing frequency was concordant to visual stimulation frequency.

**Fig 12:**
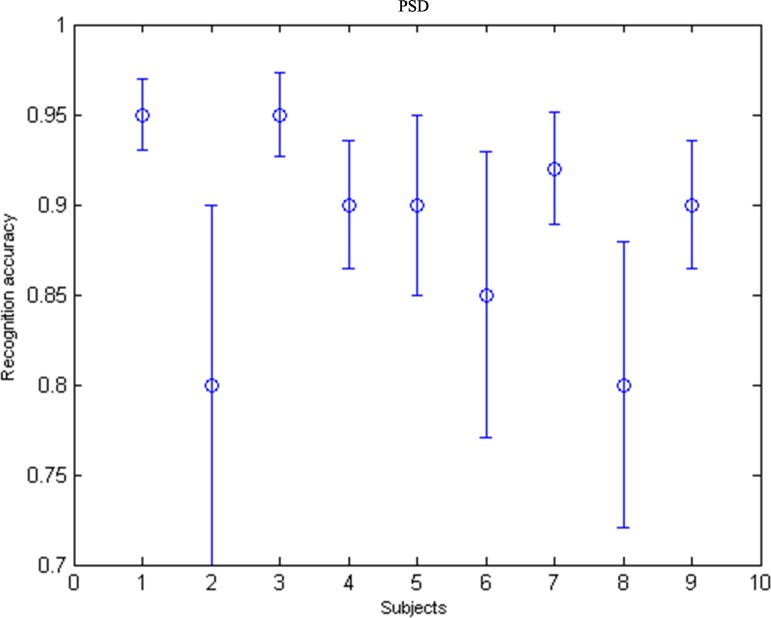
The off-line recognition accuracy error bar chart of subjects.

As shown in Fig 12, the average off-line recognition accuracy of subjects was about 88.15±6.56%, indicating that PSD-method based on the peak value of power spectral density was effective. The mean and standard deviation (SD) of the accuracy values between CCA and PSD for each subject are listed in Table 2. We can see that average recognition accuracy are very close between CCA and PSD. However, CCA accuracy results showed that individual viability had an impact on CCA. For example, the recognition accuracy of subject S2 and S8 were only 64.44% and 70.67%, while the recognition accuracy of subject S1 and S3 were 98.67% and 94.67%. Individual viability had less impact on PSD. Therefore, the PSD has better adaptability than CCA.

**Table 2:** Comparison of recognition accuracy between CCA and PSD.

Subject	CCA accuracy(mean±SD) (%)		PSD accuracy(mean±SD) (%)	
S1	98.67±1.03		93.33±4.71	
S2	64.44±5.44		82.67±3.56	
S3	94.67±2.98		89.33±3.71	
S4	86.67±4.71		88.00±5.58	
S5	88.00±8.69		92.00±7.30	
S6	89.33±7.60		86.67±10.54	
S7	93.33±5.58		93.33±4.71	
S8	70.67±7.60		81.33±13.33	
S9	93.33±4.71		86.67±5.58	
Average		86.57±5.37		88.15±6.56

## Discussion

The SSVEP-based BCI technology requires less recording electrodes for electroencephalogram (EEG) signals and no need for training. However, it usually provides stimulation through light flicking or graphic flipping, which is likely to cause visual fatigue and decrease of recognition accuracy. In order to avoid the negative impacts on the brain response caused by prolonged strong stimulation of SSVEP, this study proposed a SSMVEP stimulation method using equal-luminance colored ring-shaped checkerboard paradigm based on HVS.

### Effects of colored stimulation paradigm

To investigate the efficiency of color-enhanced visual stimulation, red-green and black-white stimulation paradigms were tested in our research. By comparing the power spectrum density of subjects between equal-luminance red-green and black-white checkerboard at 8 Hz, 11 Hz, 16 Hz, and 18 Hz, we found that equal-luminance colored stimulation evoked better SSMVEP signals with a higher SNR at the low frequency spectrum (lower than 15 Hz). The colored stimulation did not have significant enhancement effects at the middle frequency spectrum (15 Hz-25 Hz).

### Effects of stimulation frequency

Because the brain response at the low frequency spectrum is the most intensive [6], and the SNR is very low at middle frequency spectrum, therefore, the effect of colored stimulation is difficult to reflect at the middle frequency spectrum as shown in this study. Hence many stimulation frequencies have been set at the low frequency spectrum for better SSMVEP signals. In our study, equal-luminance colored stimulation was selected to determine if the method can effectively enhance brain response and increase SNR to improve BCI interactive performance. We selected 11 Hz in low frequency spectrum and 16 Hz in middle frequency spectrum to compare effects of stimulation frequency on the efficiency of stimulation paradigms. The results demonstrated that equal-luminance colored stimulation at low frequency spectrum (11 Hz) enhanced the brain response better than 16 Hz stimulation paradigm, there was no significant difference between black-white and equal-luminance red-green stimulus at middle frequency spectrum (16 Hz).

### Effects of stimulation duration

When the stimulus duration was less than 4 s, recognition accuracy of red-green checkerboard was higher than that of the black-white checkerboard. When the stimulus duration was 5 s or 6 s, there was not a statistical difference on recognition accuracy between these two stimulation paradigms. This is due to the longer stimulation duration reduced the SNR of SSMVEP response, leading to no statistical differences between two stimulation paradigms. Chi-Square test results showed that when the stimulus duration was 7 s or 8 s, the recognition accuracy of equal-luminance red-green checkerboard was higher than that of black-white checkerboard. Black-white checkerboard easily caused visual fatigue with a decrease of SNR, while equal-luminance red-green checkerboard did not cause rapid visual fatigue. Achievement of greater speed and recognition accuracy depends on improvements in signal processing, translation algorithms, and user training [23]. In our study, equal-luminance red-green checkerboard shortened the stimulation period yet produced a higher recognition accuracy suggesting that equal-luminance red-green checkerboard BCI interactive performance better than black-white checkerboard.

When the stimulation duration was longer than 6 s, subjects felt visual fatigue with a decrease of the recognition accuracy. These data suggested that the stimulation duration should be neither too short, which would fail to evoke SSMVEP, nor too long, which would cause visual fatigue.

### Signal processing and translation algorithms

In this study, a feature recognition method based on the power spectral density peak was used for signal processing. Through a spatial filtering, multi-electrode lead signals were merged into a single channel signal. Results demonstrated that the equal-luminance red-green checkerboard paradigm did not elicit obvious harmonic or sub-harmonic components. This indicates that equal-luminance red-green checkerboard paradigm may not cause cross-frequency interactions with ongoing oscillatory activity. In addition, the experiment results showed that the average recognition accuracy is about 88.15±6.56%, suggesting that the equal-luminance red-green checkerboard stimulation is a feasible and effective paradigm for SSMVEP. By comparing the recognition accuracy between CCA and PSD, we found that individual differences have less impact on PSD, suggesting that PSD has better adaptability.

## Conclusion

This study demonstrated a novel BCI paradigm based on steady-state motion visual evoked potential (SSMVEP). The new paradigm integrated equal-luminance ring-shaped checkerboard into visual stimulation paradigms. Results demonstrated that equal-luminance red-green checkerboard paradigm had the lower fatigue characteristics, less visual discomfort, and significantly stronger responses than black-white checkerboard paradigm. The signal processing and translation algorithms for feature recognition are feasible and effective. The equal-luminance red-green checkerboard paradigm evoked SSMVEP with a better SNR than black-white checkerboard at low frequency spectrum (below 15 Hz). The recognition accuracy was 88.15±6.56%.

## Supporting Information

S1 File The power spectral density of 9 subjects in the 8 Hz、11 Hz、16 Hz、18 Hz. (XLS)

S2 File The recognition accuracy of 9 subjects under different stimulation duration. (XLS)

S1 Video The movement patterns of the checkerboard. Through video, readers can better understand the process of the contraction and expansion of the equal-luminance red-green checkerboard.
(AVI)

## References

[1] Miner LA, McFarland DJ, Wolpaw JR. Answering questions with an EEG-based brain-computer interface (BCI). Archives of Physical Medicine and Rehabilitation. 1998 9;79(9):1029–1033. 9749678 10.1016/s0003-9993(98)90165-4

[2] Yin E, Zeyl T, Saab R, Chau T, Hu D, Zhou Z. A hybrid brain-computer interface based on the fusion of P300 and SSVEP scores. IEEE Transactions on Neural Systems and Rehabilitation Engineering. 2015 7;23(4):693–701. 10.1109/TNSRE.2015.2403270 25706721

[3] Vidal JJ. Toward direct brain-computer communication. Annual review of Biophysics and Bioengineering. 1973 6;2(1):157–180. 10.1146/annurev.bb.02.060173.001105 4583653

[4] Yin E, Zhou Z, Jiang J, Yu Y, Hu D. A dynamically optimized SSVEP brain-computer interface (BCI) speller. IEEE Transactions on Biomedical Engineering. 2015 6;62(6):1447–1456. 10.1109/TBME.2014.2320948 24801483

[5] Snowden RJ, Freeman TCA. The visual perception of motion. Current Biology. 2004 10;14(19):R828–R831. 10.1016/j.cub.2004.09.033 15458658

[6] Vialatte FB, Maurice M, Dauwels J, Cichocki A. Steady-state visually evoked potentials: focus on essential paradigms and future perspectives. Progress in Neurobiology. 2010 4;90(4):418–438. 10.1016/j.pneurobio.2009.11.005 19963032

[7] Guo F, Hong B, Gao X, Gao S. A brain–computer interface using motion-onset visual evoked potential. Journal of Neural Engineering. 2008 11;5(4):477–485. 10.1088/1741-2560/5/4/011 19015582

[8] Hong B, Guo F, Liu T, Gao X, Gao S. N200-speller using motion-onset visual response. Clinical neurophysiology. 2009 9;120(9):1658–1666. 10.1016/j.clinph.2009.06.026 19640783

[9] Hammond P, Mouat GSV, Smith AT. Motion after-effects in cat striate cortex elicited by moving gratings. Experimental Brain Research. 1985 10;60(2):411–416. 4054284 10.1007/BF00235938

[10] Xie J, Xu G, Wang J, Zhang F, Zhang Y. Steady-state motion visual evoked potentials produced by oscillating newton’s rings: implications for brain-computer interfaces. Plos one. 2012 6;7(6):e39707 10.1371/journal.pone.0039707 22724028 PMC3378577

[11] Xie J, Xu G, Wang J, Zhang S, Zhang F, Li Y, et al Addition of visual noise boosts evoked potential-based brain-computer interface. Scientific reports. 2014 5;4(4953). 10.1038/srep04953 PMC4021798 24828128

[12] Bönigk W, Altenhofen W, Müller F, Dose A, Illing M, Molday Rs, et al Rod and cone photoreceptor cells express distinct genes for cGMP-gated channels. Neuron. 1993 5;10(5):865–877. 7684234 10.1016/0896-6273(93)90202-3

[13] Mishkin M, Ungerleider LG. Contribution of striate inputs to the visuospatial functions of parieto-preoccipital cortex in monkeys. Behavioural brain research. 1982 9;6(1):57–77. 7126325 10.1016/0166-4328(82)90081-x

[14] Delon-Martin C, Gobbele R, Buchner H, Haug BA, Antal A, Darvas F, et al Temporal pattern of source activities evoked by different types of motion onset stimuli. Neuroimage. 2006 7;31(4):1567–1579. 10.1016/j.neuroimage.2006.02.013 16580846

[15] Ales JM, Norcia AM. Assessing direction-specific adaptation using the steady-state visual evoked potential: Results from EEG source imaging. Journal of Vision. 2009 7;9(7):8–8. 10.1167/9.7.8 19761323

[16] Stockman A, Macleod DIA, Johnson NE. Spestral sensitivities of the human cones. Journal of the Optical Society of America A Optics Image Science and Vision. 1993 12;10(12):2491–2521. 10.1364/josaa.10.002491 8301403

[17] Stockman A, Sharpe LT, Fach C. The spectral sensitivity of the human short-wavelength cones. Vision Research. 1999 8;39(17):2901–2927. 10492818 10.1016/s0042-6989(98)00225-9

[18] Stockman A, Sharpe LT. The spectral sensitivities of the middle-and long-wavelength-sensitives cones derived from measurements in observers of known genotype. Vision Research. 2000 6;40(13):1711–1737. 10814758 10.1016/s0042-6989(00)00021-3

[19] McCann JJ, McKee SP, Taylor TH. Quantitative Studies in Retinex Theory-Comparison Between Theoretical Predictions and Obaerver Responses to Color Mondrian Experiments. Vision Research. 1976 2;16(5):445–458. 941426 10.1016/0042-6989(76)90020-1

[20] Alkan A, Kiymik MK. Comparison of AR and Welch Methods in Epileptic Seizure Detection. Journal of Medical Systems. 2006 12;30(6):413–419. 17233153 10.1007/s10916-005-9001-0

[21] Wolpaw JR, McFarland DJ. Multichannel EEG-based brain-computer communication. Electroencephalography and clinical Neurophysiology. 1994;90(6):444–449. 7515787 10.1016/0013-4694(94)90135-x

[22] Peters BO, Pfurtscheller G, Flyvbjerg H. Mining multi-channel EEG for its information content: an ANN-based method for a brain-computer interface. Neural Network. 1998;11(7):1429–1433. 10.1016/s0893-6080(98)00060-4 12662759

[23] Wolpaw JR, Birbaumer N, Heetderks WJ, McFarland DJ, Peckham PH, Schalk G, et al Brain-computer interface technology: a review of the first international meeting. IEEE Trans Rehabil Eng. 2000;8(2): 164–173. 10896178 10.1109/tre.2000.847807

